# Supplementary material for: Microfiber-Patterned Versatile Perfusable Vascular Networks
Source: Micromachines (Basel). 2023 Dec 1;14(12):2201. doi: 10.3390/mi14122201 (PMC10745573; doi:10.3390/mi14122201)
Supplement: Supplementary file 1 [file micromachines-14-02201-s001.zip › Supplementary Materials.pdf]

micromachines

Supplementary Materials

Microfiber-Patterned Versatile Perfusable Vascular Networks

Ye Tian [1,2,*] and Liqiu Wang [3,*]

[1] College of Medicine and Biological Information Engineering, Northeastern University, Shenyang 110169, China
[2] Foshan Graduate School of Innovation, Northeastern University, Foshan 528300, China
[3] Department of Mechanical Engineering, The Hong Kong Polytechnic University, Hong Kong, China
* Correspondence: tianye@bmie.neu.edu.cn (Y.T.); liqiu.wang@polyu.edu.hk (L.W.)

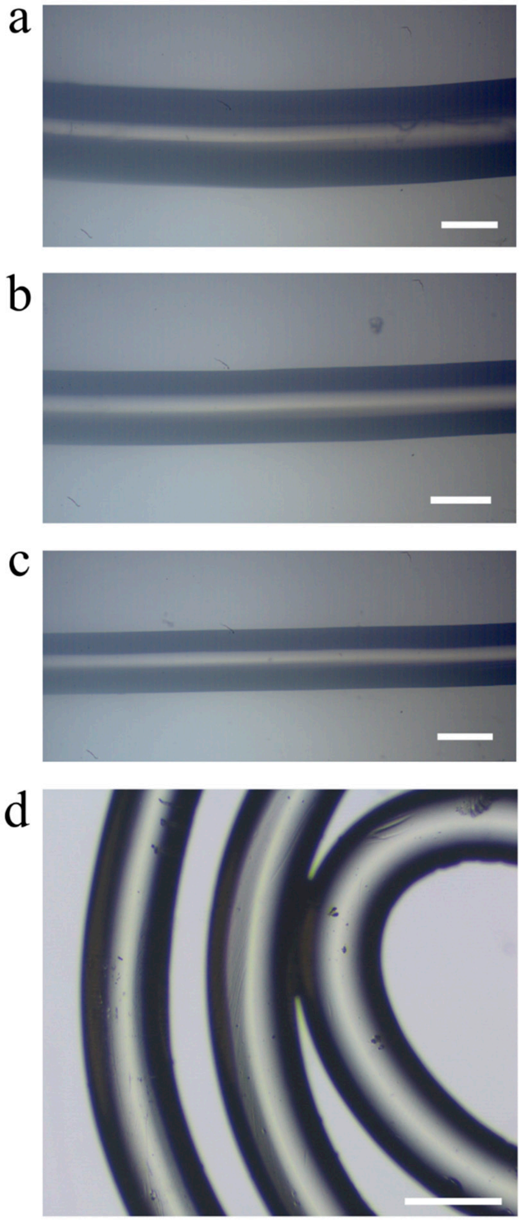

Figure S1. Microfibers fabricated via microfluidics with different diameters. We use simple capillary-based microfluidic device to generate calcium alginate microfibers with different diameters: (**a**) 864 μm, (**b**) 619 μm, (**c**) 555 μm and (**d**) 442 μm. Scale bar, 500 μm.

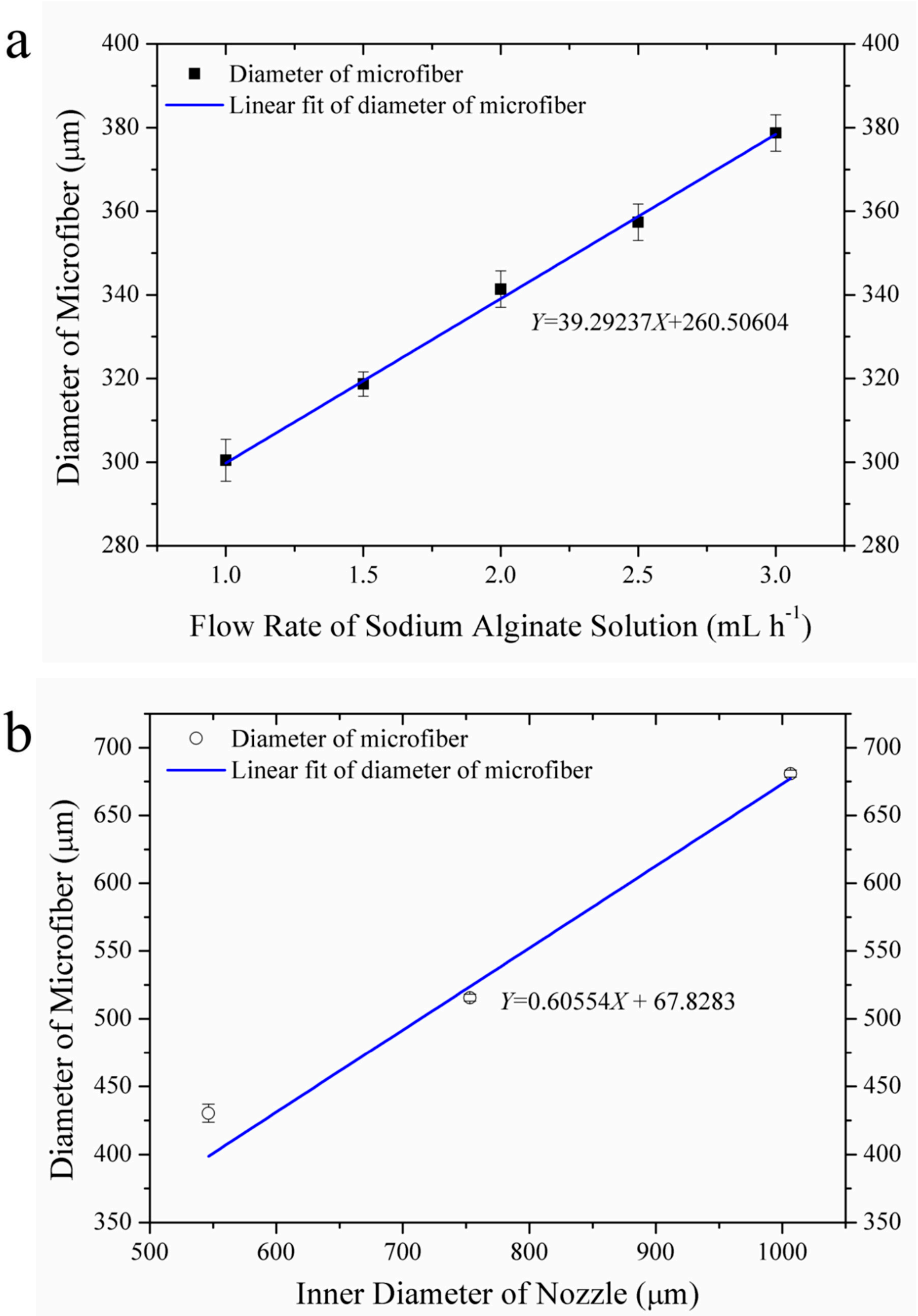

Figure S2. Tunable diameters of microfibers. Through changing the flow rate of 4 wt% sodium alginate solution and the diameter of nozzle, respectively, we can tune the diameter of microfibers conveniently. (**a**) A plot of the diameter of microfiber against the flow rate of 4 wt% sodium alginate solution. (**b**) A plot of the diameter of microfiber against the inner diameter of nozzle under

the flow rate of 4 wt% sodium alginate solution, 3 mL h^{-1}. All error bars in (**a**,**b**) indicate the standard deviations over three independent measurements.

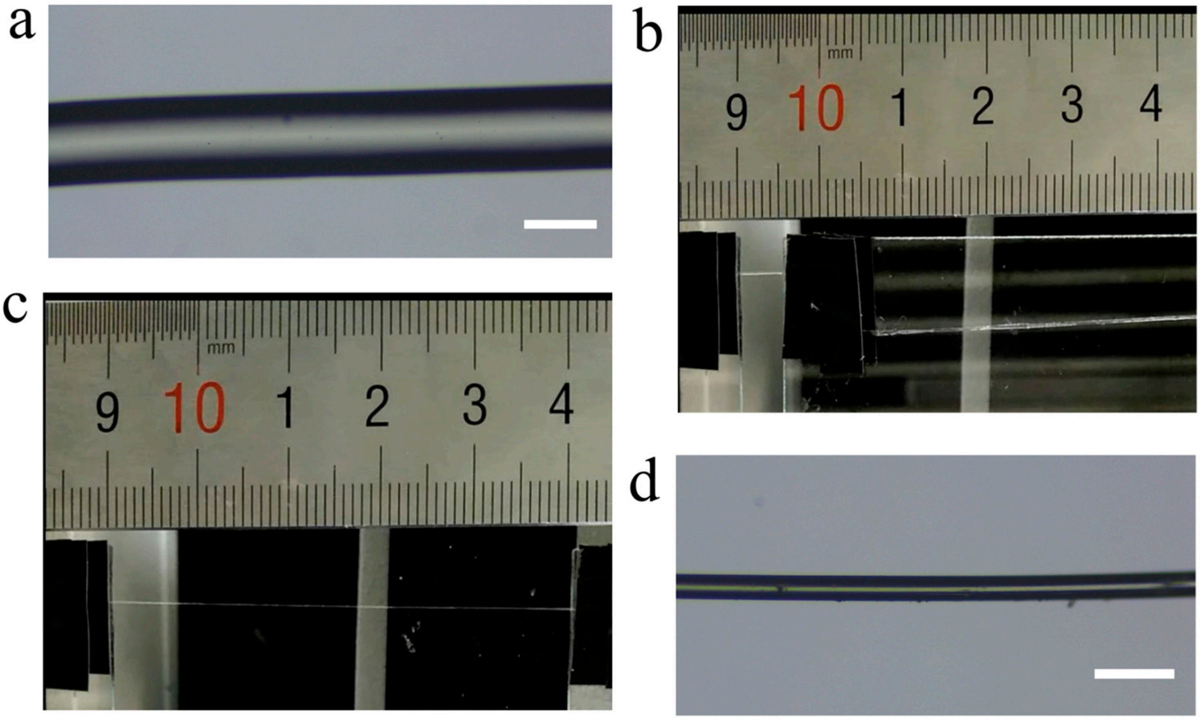

Figure S3. Mechanical strength of microfiber. (**a**) Experimental microfiber sample. (**b**) Original microfiber sample with the length of 0.5 cm. (**c**) The microfiber in (**b**) was stretched to 5 cm. (**d**) Microfiber after tensile test. Scale bar, 400 μm.

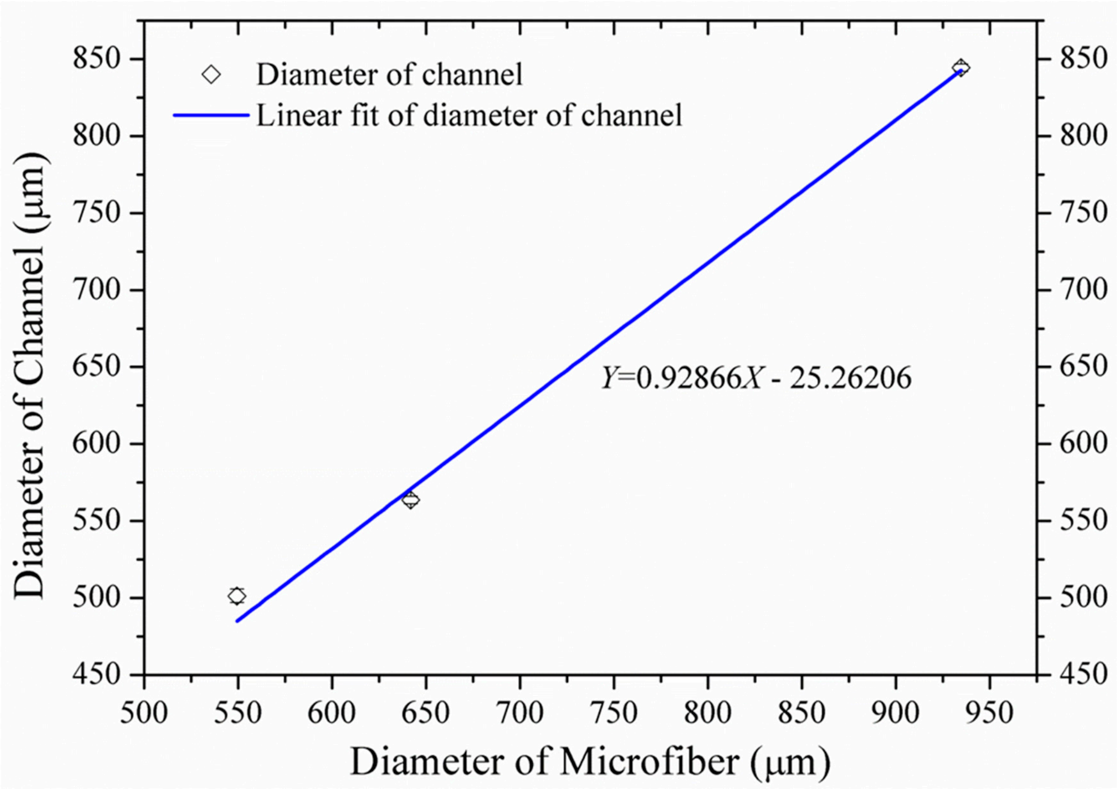

Figure S4. A plot of the diameter of channel against the diameter of microfiber. Through tuning the diameter of microfiber, we can fabricate the vascular network with a broad range of channel diameters. All error bars indicate the standard deviations over three independent measurements.

a b c d e f

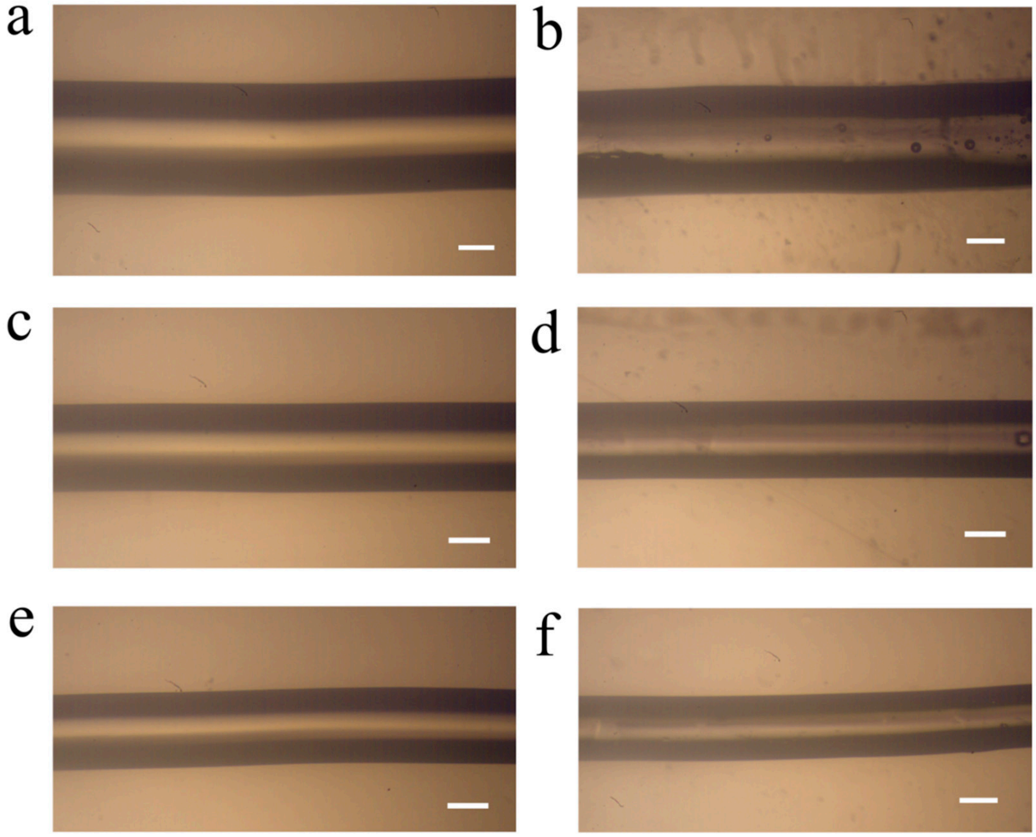

Figure S5. Microfibers and corresponding channels. (**a**) Microfiber with the diameter of 934.677 μm. (**b**) The channel with the diameter of 844.462 μm corresponding to (**a**). (**c**) Microfiber with the diameter of 641.800 μm. (**d**) The channel with the diameter of 563.572 μm corresponding to (**c**). (**e**) Microfiber with the diameter of 549.342 μm. (**f**) The channel with the diameter of 501.333 μm corresponding to (**e**). Scale bar, 300 μm.

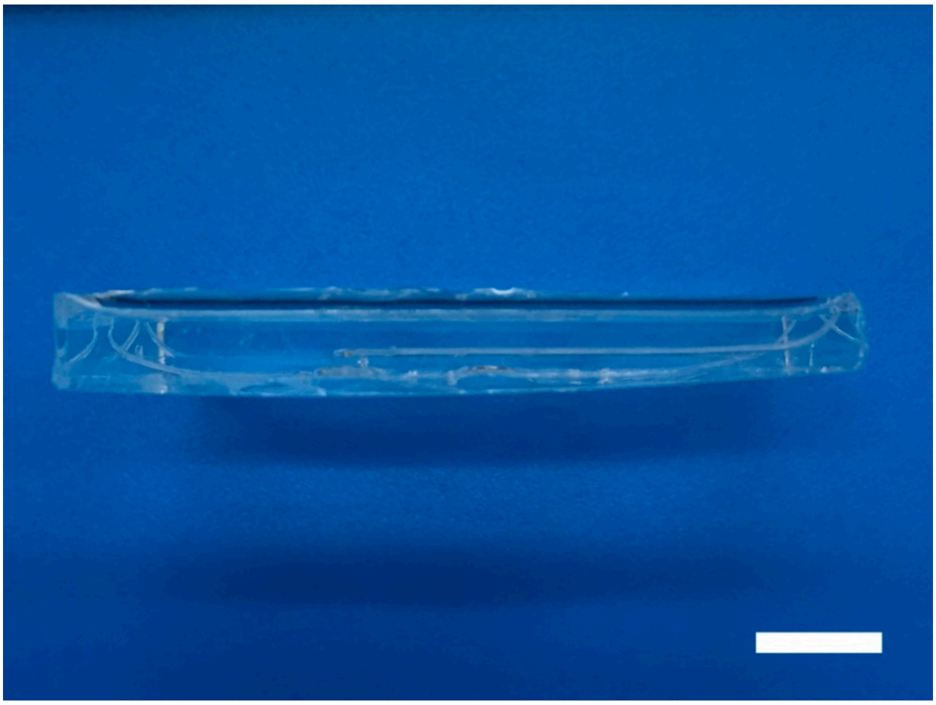

Figure S6. Side view of two-layered vascular network. The side view of the two-layered vascular network without interconnection demonstrates the two independent layers in this chip. Scale bar, 10 mm.

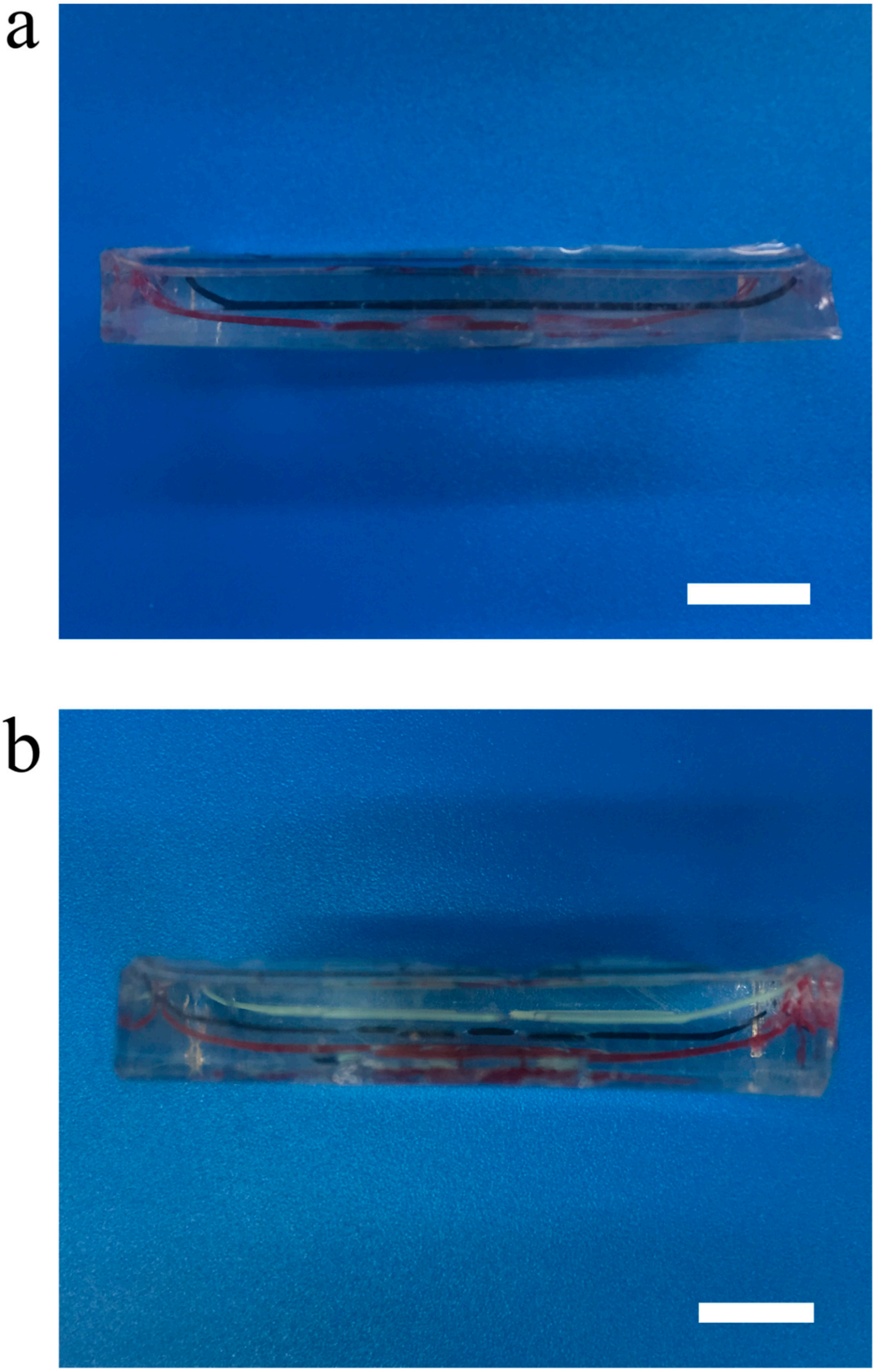

Figure S7. Side views of perfused multilayered vascular networks. (**a**) The side view of two-layered vascular network filled with two dye-solutions. (**b**) The side view of three-layered vascular network filled with three dye-solutions. Scale bar, 10 mm.

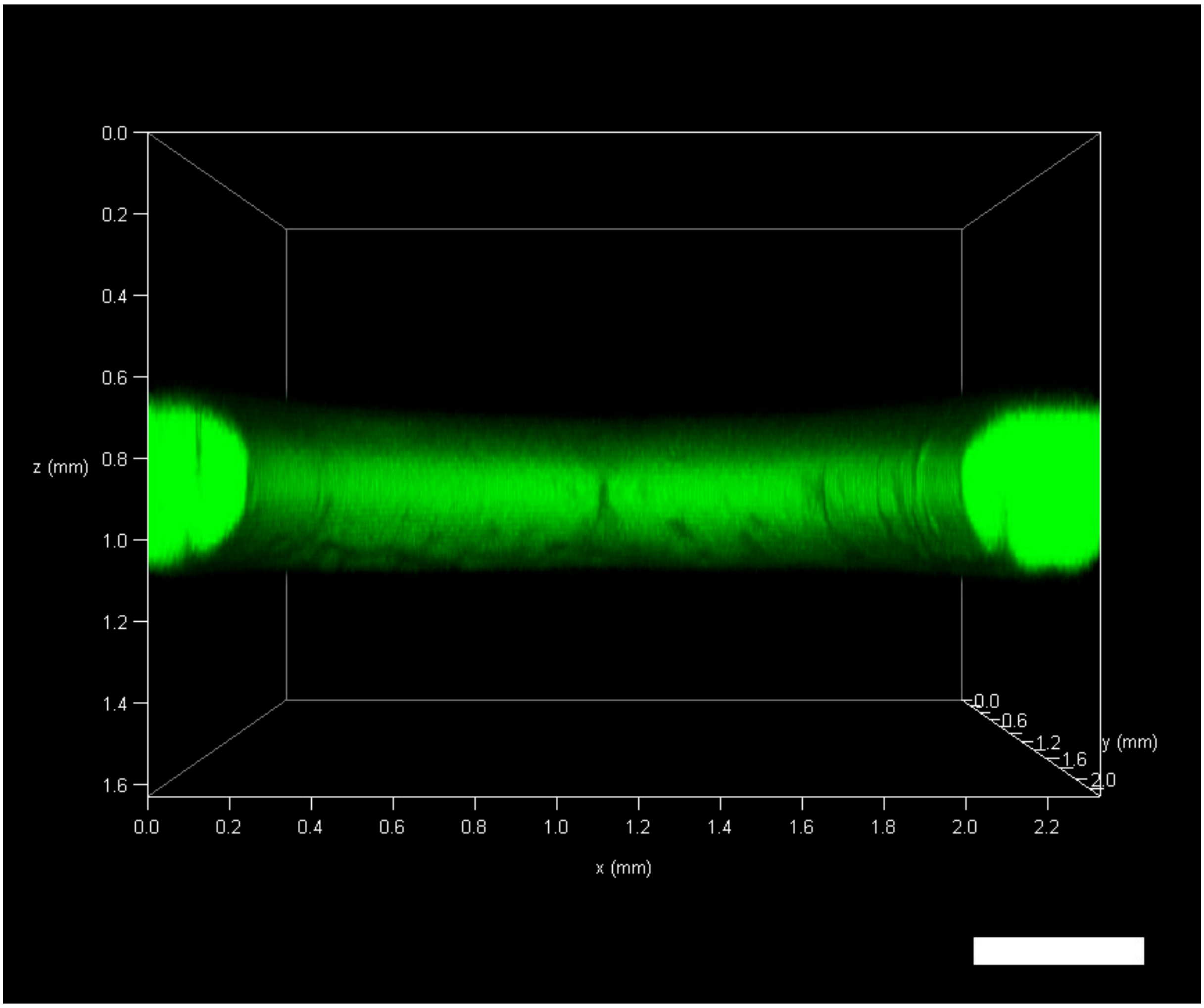

Figure S8. Side view of 3D reconstructed biomicromolecule distribution. The side view of 3D biomicromolecule distribution in the 2D vascular network was shown via confocal microscope 3D reconstruction method. Scale bar, 500 μm.

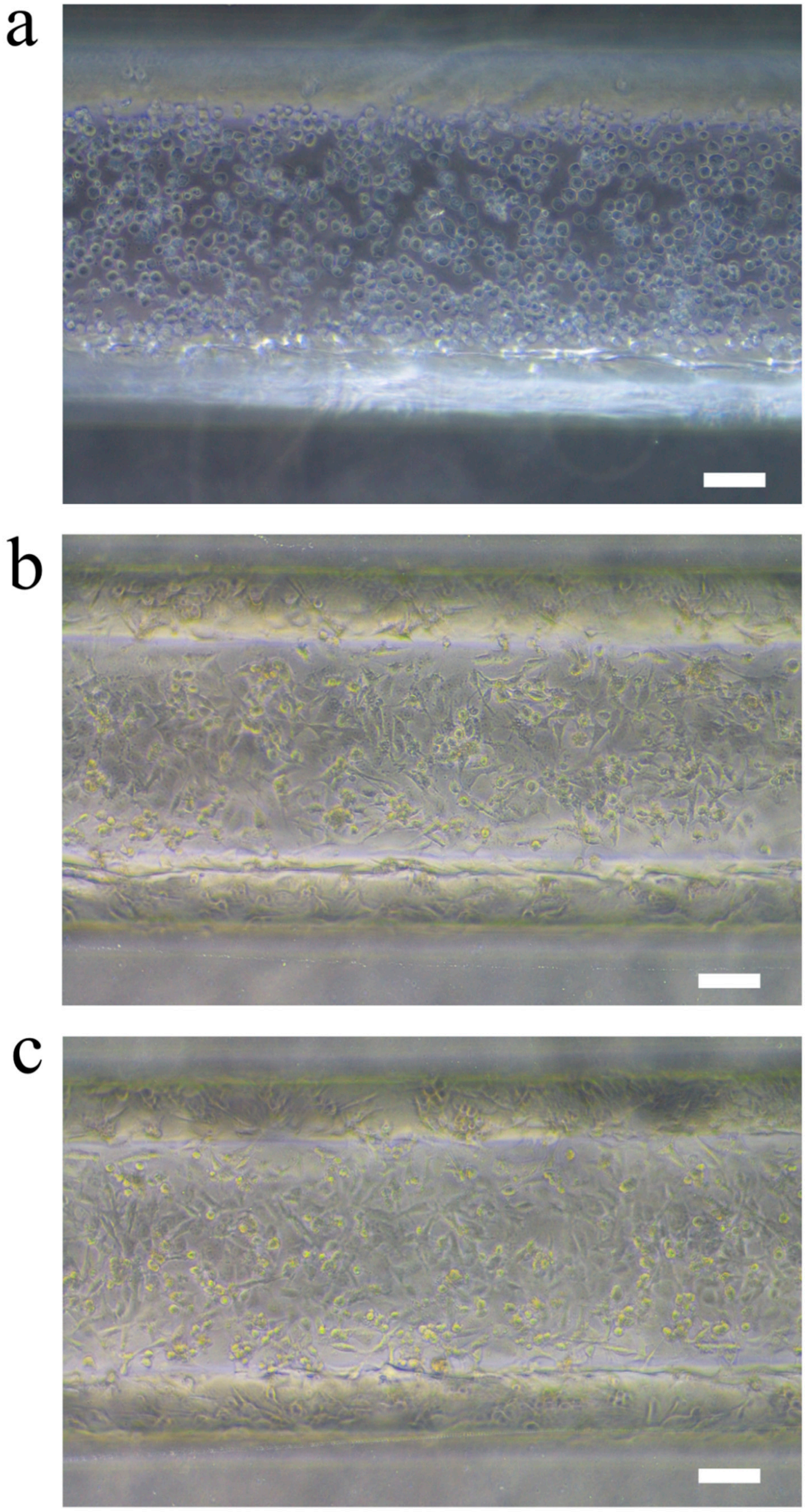

Figure S9. Time sequence of HUVECs culture. (**a**) HUVECs just were perfused into PDMS hollow fiber (0 h). (**b**) HUVECs were cultured after 24 h. (**c**) HUVECs were cultured after 48 h. Scale bars, 100 μm.

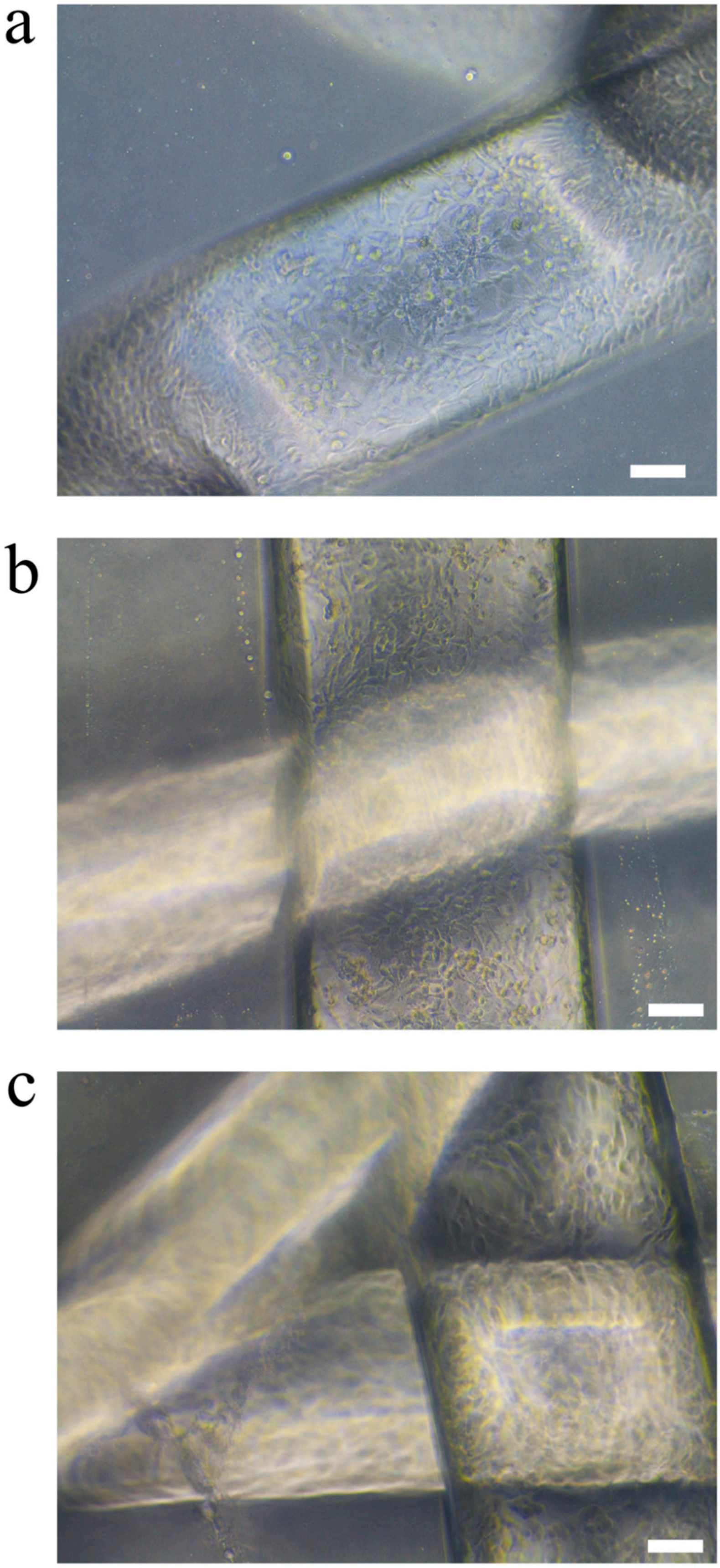

Figure S10. Optical images showing the good cell status. Optical images show the good cell status in (**a**) 3D helicoid vascular network, (**b**) two-layered vascular network and (**c**) three-layered vascular network. Scale bars, 100 μm.

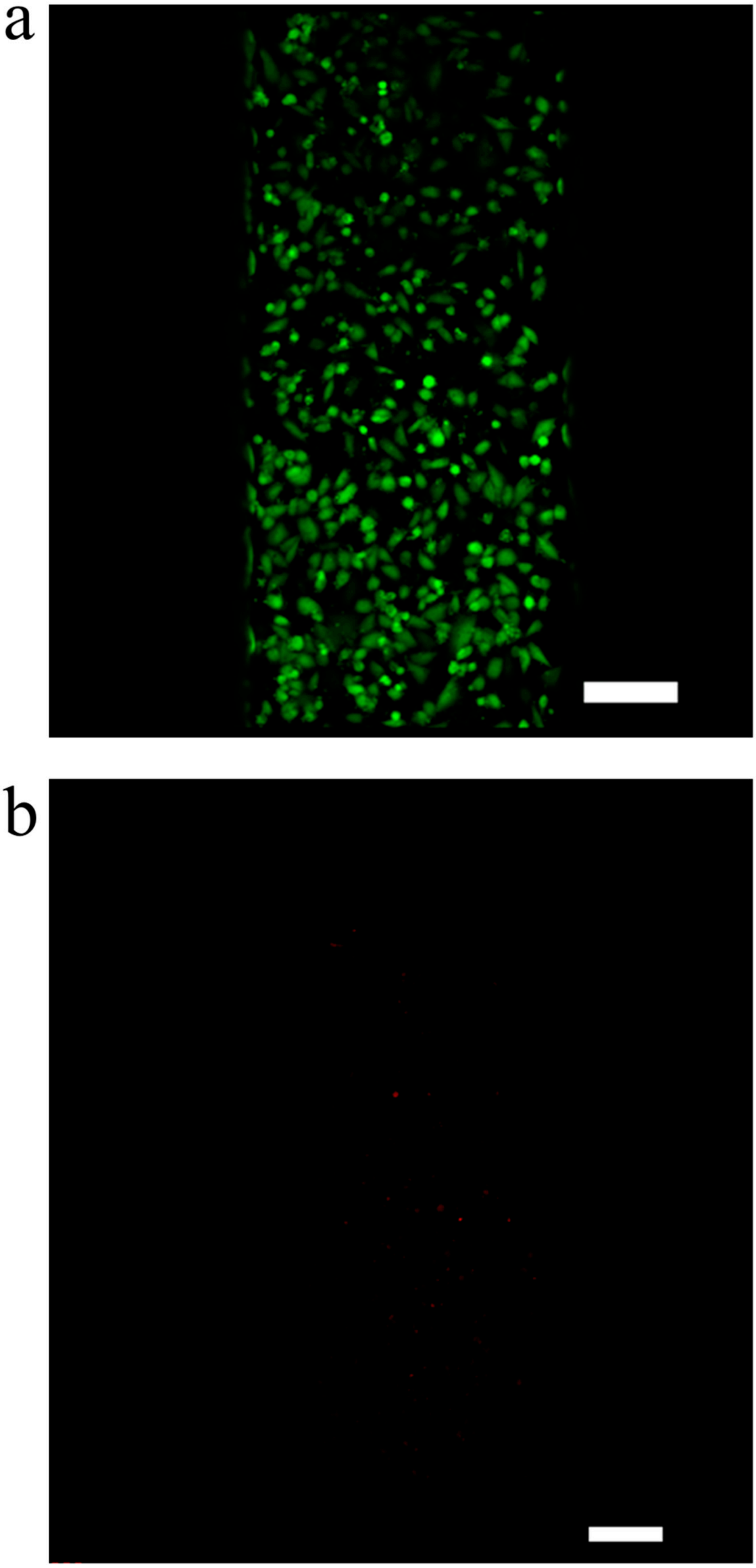

Figure S11. Live/dead staining for HUVECs cultured for 4 days. Live/dead staining of HUVECs on the interior surface of vascular networks confirms the cell viability after 4 days culture. (**a**) HUVECs were stained with Calcein-AM. (**b**) HUVECs were stained with PI. Scale bars, 150 μm.

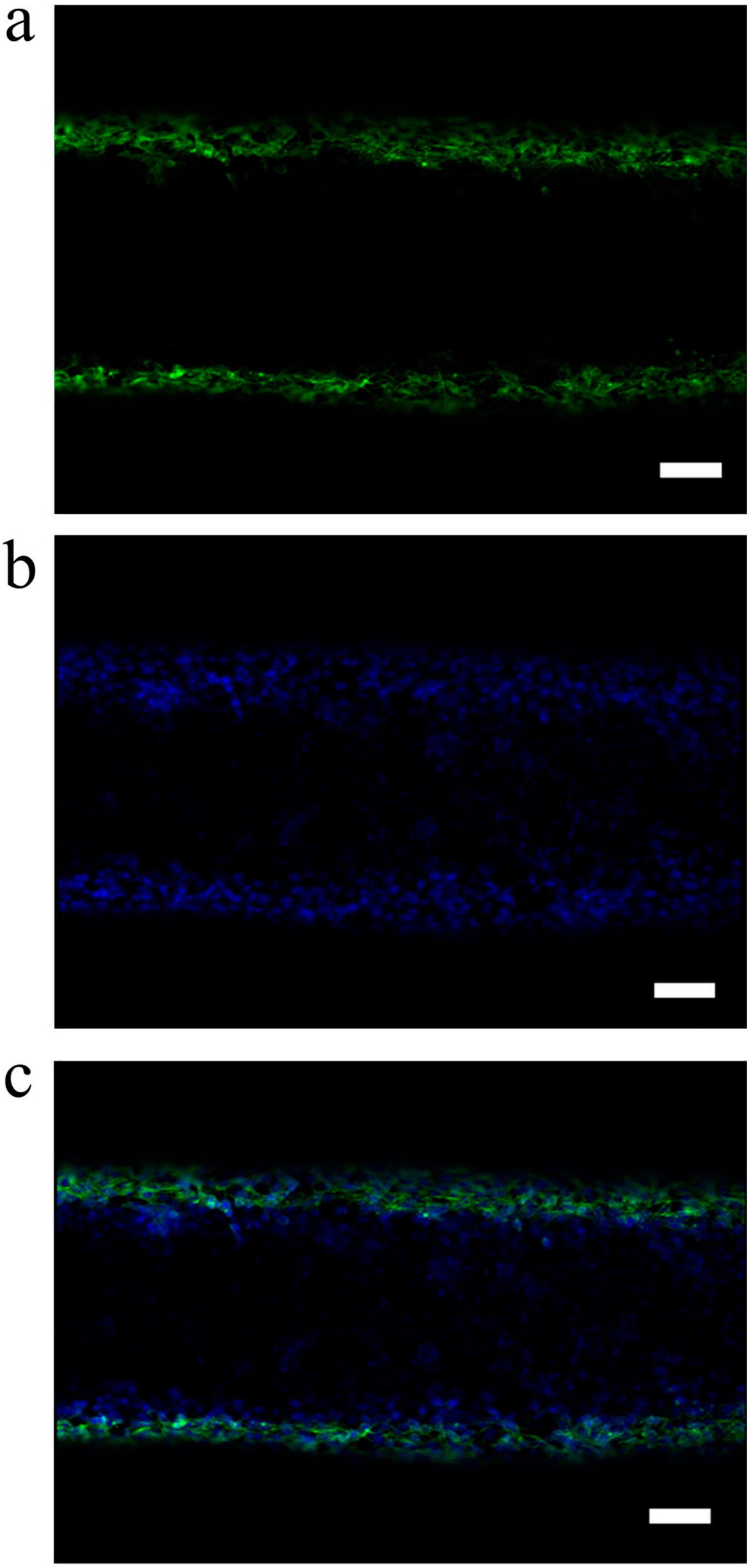

Figure S12. Stained HUVECs at the middle of vascular network. Fluorescence microscopic images (**a**) ALEXA 488 Phalloidin (green) and (**b**) DAPI (blue) staining and (**c**) superimposed image show the good endothelial monolayer at the middle of vascular network. Scale bars, 100 µm.

Legends for Supporting Information, Movie 1 to Movie 4:

Supporting Information, Movie S1. Microfiber can be pulled out directly. After the PDMS pre-polymerization, the microfiber can be pulled out directly from the pre-polymerized PDMS due to the strong mechanical strength of microfiber and the separation between the PDMS vascular network chip and the microfiber.

Supporting Information, Movie S2. Interconnection of interconnected vascular network. The dye solution can be perfused into the other layered channel from the top layered channel through the interconnection of interconnected vascular network.

Supporting Information, Movie S3. Perfusable ability of 2D vascular network. The red dye solution flowed smoothly through the 2D vascular networks from the inlet to outlet over time.

Supporting Information, Movie S4. Perfusable ability of 3D vascular network. The red dye solution flowed smoothly through the 3D vascular networks from the inlet to outlet over time.
